# Combined Immediate‐Release and Extended‐Release Formulation of Sodium Valproate Provides Stable Plasma Levels for Inhibition of Histone Deacetylation

**DOI:** 10.1002/cpdd.1555

**Published:** 2025-05-27

**Authors:** Nikhil Ahuja, Susanna Kääriäinen, Zsófia Lovró, Mia Lundblad, Kristina Drott, Elsa Lilienberg, Marica T. Engström, Karla Saukkonen, Mika Scheinin

**Affiliations:** ^1^ Clinical Research Services Turku, CRST Oy Finland; ^2^ Valcuria AB (wholly owned subsidiary of Respiratorius AB) Sweden; ^3^ SDS Life Science AB (part of Cytel Inc.) Sweden; ^4^ Institute of Biomedicine University of Turku Finland

**Keywords:** diffuse large B‐cell lymphoma, formulation, histone deacetylase, pharmacokinetics, sodium valproate

## Abstract

A modified controlled‐release sodium valproate formulation (VAL001, test) was compared with an approved enteric‐coated tablet formulation (Absenor, reference). Pharmacokinetics and safety/tolerability were evaluated in healthy subjects to bridge with positive efficacy results from an early‐phase patient trial of valproate in combination with chemotherapy in diffuse large B‐cell lymphoma. In Part I (n = 12), both formulations were administered as single doses (30 mg/kg) in a randomized crossover fashion. Equivalent exposures (area under the plasma concentration–time curve) for total and free valproate were observed under fasted conditions. Intake with food delayed the absorption of valproate from the test formulation, with no impact on AUC. In Part II (n = 27), both formulations were administered over 3 consecutive days, at 30 mg/kg twice daily (test) or 20 mg/kg 3 times daily (reference). Similar steady‐state levels were observed, but fluctuation was less with the test product (23% vs. 47%, *P* = .0102). Inhibition of histone deacetylase activity was evidenced by increased levels of acetylated H3K9 in peripheral blood mononuclear cells. No serious or severe adverse events were observed. The novel capsule formulation of valproate, containing a combination of immediate‐release granules and extended‐release pellets, appears to have suitable pharmacokinetic properties for cancer treatments aiming for histone deacetylase inhibition.

Many forms of cancer involve alterations in DNA methylation and modification of histone proteins. Acetylation is an important posttranslational histone modification mechanism that modulates chromatin remodeling and hence gene expression. It is controlled by a balance of opposing activities of histone acetyltransferases and histone deacetylases (HDACs). In recent years, HDACs have emerged as an important target in cancer therapeutics. Valproic acid (valproate) is an old antiepileptic drug that was identified in 2001 as an HDAC inhibitor.^1^, ^2^ It has therefore been investigated as a likely therapeutic candidate in oncology, either alone or in combination with other forms of therapy.

There is limited clinical evidence for its efficacy as monotherapy, for example, in neuroendocrine tumors, acute myeloid leukemia, and low‐risk myelodysplastic syndrome.^3^, ^4^, ^5^ As part of combination therapies, valproate has shown promising antitumor activity in several preclinical and early clinical studies.^6^, ^7^, ^8^, ^9^, ^10^ Its proposed mode of action in combination therapies is that inhibition of HDAC activity would have synergistic effects with cancer chemotherapy by at least 2 mechanisms: first, by tilting the balance between oncogenes and tumor suppressor genes in favor of tumor suppressors, and second, by altering the sensitivity to DNA‐damaging chemotherapy through modulation of chromatin structure.^8^, ^11^ Valproate therefore has the potential to be a part of treatment regimens in various cancers due to its HDAC inhibitory activity.

It was recently shown that valproate may add benefit to standard treatment with R‐CHOP—that is, rituximab, cyclophosphamide, doxorubicin, vincristine, and prednisone—in patients with diffuse large B‐cell lymphoma (DLBCL).^10^ The addition of valproate to standard R‐CHOP treatment significantly increased the overall survival of patients with DLBCL, compared to a matched cohort of patients from the Swedish Lymphoma Registry who had received standard treatment only (ie, no valproate). Moreover, in the study by Drott et al.,^10^ the maximally tolerated dose of valproate was established as 60 mg/kg/day. The formulation of valproate that was evaluated in this Phase I/IIa trial^10^ was similar to the reference formulation used in the current study, that is, conventional enteric‐coated tablets.

The current study was a 2‐part, open‐label, Phase I trial designed to investigate the pharmacokinetics (PK) as well as safety and tolerability of a novel modified‐release formulation of sodium valproate (test formulation) in healthy subjects. Part I compared the PK characteristics of single 30‐mg/kg doses of the test formulation with those of conventional enteric‐coated immediate‐release tablets (reference formulation) in the fasted state and also evaluated the effect of a high‐fat, high‐calorie meal on the absorption of valproate from the test product. Part II of the study evaluated and compared the PK characteristics of the 2 products upon repeated dosing. Also, valproate‐induced increases in histone acetylation in peripheral blood mononuclear cells (PBMCs) were compared after repeated dosing of the test and reference formulations.

## Methods

The study was approved on October 28, 2021, by the National Committee on Medical Research Ethics (TUKIJA), Helsinki, Finland, and subsequently by the Finnish Medicines Agency Fimea. Informed consent was obtained from all study subjects before participation. The study was performed at Clinical Research Services Turku, CRST Oy, in Finland, with a main site located in Turku and a satellite site located in Helsinki.

Healthy male and female subjects, 18‐64 years of age, with a body mass index of 18.5‐30.0 kg/m^2^ were recruited for the study. Eligible participants were to be capable of providing valid independent informed consent. Medical history, physical examination results, electrocardiogram (ECG), and laboratory results (clinical chemistry, hematology, urine dipstick analysis) were evaluated to exclude any clinically significant medical conditions and use of illicit drugs. An audiogram was recorded to exclude subjects with impaired hearing and to provide baseline information for safety follow‐up. Female subjects were required to be surgically sterilized or postmenopausal for 1 year or more. Female subjects of childbearing potential could also be included if it was their preferred and permanent lifestyle to abstain from heterosexual relationships, and if they agreed to continue such abstinence during their study participation. Pregnancy was ruled out with repeated pregnancy tests.

### Study Design

The study employed an open‐label, randomized design. Part I of the study compared the PK, safety, and tolerability of a single 30‐mg/kg dose of the test formulation with the same dose of the reference product, under fasting and fed conditions. Part II compared 3‐day dosing regimens of the test and reference products at approximately 60 mg/kg daily doses. The individual doses were approximated to the nearest available capsule/tablet strengths of the test and reference formulations.

In Part I, participants were randomly allocated to 1 of 3 treatment sequences in a 3‐treatment, 3‐period crossover design (Figure S1). The participants received single doses (approximately 30 mg/kg) of the test formulation under fasted (10 hours or more) conditions (treatment A) or under fed conditions (treatment B), or the reference formulation under fasted conditions (treatment C). The doses were separated by washout periods of 10‐21 days. Subjects remained at the clinical site for at least 24 hours after each dose intake and returned to the site for additional PK sampling and safety evaluations on Day 2 (32 hours), Day 3 (48 hours), Day 4 (72 hours), and Day 5 (96 hours) after dosing. Samples for safety laboratory testing were collected just before dosing and 24 hours after each dose. An ECG was recorded just before dosing and 3 and 24 hours after each dose. Vital signs were recorded just before dosing and 3, 12, and 24 huours after each dose. Venous blood samples for PK concentration analysis of valproate were collected before dosing and at 0.5, 1, 2, 3, 3.5, 4, 4.5, 5, 5.5, 6, 7, 8, 9, 10, 12, 24, 32, 48, 72, and 96 hours after dosing. Just before dosing in the fed condition, a standardized high‐fat, high‐calorie meal was served. An end‐of‐study visit (including a physical examination, laboratory tests, and a repeat audiogram) took place 14‐21 days after the last dose.

The main objectives of Part I were to determine and compare the bioavailability and PK of single doses of the test and reference formulations under fasted conditions and to assess the influence of food intake on the PK of the test formulation. The tolerability of the 2 formulations was also compared. In total, 13 healthy subjects were randomized, and 12 subjects completed Part I of the trial, 4 in each sequence (Figure S1).

Part II followed a parallel‐group design where 27 subjects were randomized to receive the test product (30 mg/kg twice daily, n = 13) or the reference product (20 mg/kg 3 times daily, n = 14) for 3 consecutive days, resulting in total doses of 180 mg/kg, administered as 60 mg/kg/day under fed conditions (ie, a standard breakfast, lunch, dinner, or snack preceded each dose intake). Subjects remained at the clinical site until the evening of Day 4. A safety evaluation was performed before discharge. The subjects returned to the site for additional PK sampling and safety evaluations on Day 5 (96 hours after the first dose), Day 6 (120 hours), Day 7 (144 hours), and Day 8 (168 hours after the first dose). Samples for safety laboratory testing were collected just before the start of dosing, in the mornings of Days 2 and 3, and before discharge on Day 4. ECG was recorded just before the start of dosing, 3 hours after each morning dose, and before discharge. Vital signs were recorded just before the start of dosing, 3 hours after each morning dose, 12 hours after the first dose, in the mornings of Days 2 and 3, and before discharge on Day 4. Venous blood samples for PK concentration analysis of valproate were collected before the start of dosing, frequently during the dosing period, and until 168 hours after the first dose (see Figure 2 for time points). An end‐of‐study visit (including a physical examination, laboratory tests, and a repeat audiogram) took place 14‐21 days after the last dosing. All 27 subjects completed the study.

### Study Treatments

The investigational medicinal products used were VAL001 capsules (test product; Valcuria AB, Lund, Sweden), containing 250 mg of sodium valproate (containing 75% of immediate‐release granules and 25% of extended‐release pellets), and Absenor immediate‐release enteric‐coated tablets (reference product; Orion Pharma, Espoo, Finland), containing 100 or 500 mg of sodium valproate.

### Bioanalysis

Quantitative analysis of total and free valproate in plasma samples was performed at the Bioanalytical Laboratory of the University of Turku. To determine total valproate concentrations in plasma, the frozen plasma samples were first thawed unassisted at room temperature, and sample preparation was carried out using protein precipitation and phospholipid removal with Ostro sample preparation plates (Waters Co.). For the determination of protein‐unbound (free) valproate concentrations, plasma aliquots of 950 µL were pipetted into Centrifree Ultrafiltration Centrifugal Filters (Merck KGaA) and centrifuged for 15 minutes at 1900 g until approximately 100 µL of ultrafiltrate was recovered for subsequent processing with Ostro plates. Deuterated valproate (valproate‐D_6_) was used as the internal standard in the analysis.

Liquid chromatography–tandem mass spectrometry analyses were performed with a validated high‐performance liquid chromatography–tandem mass spectrometry method on an Exion liquid chromatography system coupled to an AB Sciex QTRAP 6500+ triple‐quadrupole detector. Separations were performed with a Phenomenex Kinetex C18, 2.1 × 100‐mm, 2.6‐µm column coupled with a Phenomenex SecurityGuard ULTRA C18 2.1 × 4‐mm guard column. The mobile phase consisted of 0.1% formic acid in water (A) and 0.1% formic acid in acetonitrile (B) with a constant flow rate of 0.2 mL/min with the following gradient: 0‐0.5 minutes, 40% B in A; 0.5‐2 minutes, 40%‐50% B in A (linear gradient); 2‐4 minutes, 50% B in A; 4‐4.25 minutes, 50%‐90% B in A (linear gradient); 4.25‐6.25 minutes, 90% B in A; 6.25‐6.50 minutes, 90%‐40% B in A (linear gradient) and 6.5‐8.5 minutes, 40% B in A. The column oven temperature was set to +40°C and the autosampler temperature to +4°C. Injection volume was 10 µL. Mass spectrometric detection was carried out with negative Turbo Ion Spray (TIS) ionization and pseudo multiple reaction monitoring mode. TIS temperature was +500°C. The nebulizer gas (Gas 1) setting was 60 and the turbo gas (Gas 2) setting was 70 for both analytes. The TIS voltage setting was −4500 V. The declustering potential was −35 V and the collision energy was −5 V. The entrance potential was set to −10 V and the collision cell exit potential was set to −20 V. The pseudo–multiple reaction monitoring transitions were m/z 143.0 → m/z 143.0 for valproate and m/z 149.0 → m/z 149.0 for valproate‐D_6_. The dwell time for both molecules was 200 ms.

The total number of samples analyzed for total valproate was 1753. Incurred samples reanalysis was performed for 138 samples. The samples were analyzed in 30 analysis batches. In each study sample batch, quality control (QC) samples were analyzed to further validate the analytical method. The interassay precision (coefficient of variation [CV%]) for the accepted QC samples in the batches was 3.0%‐6.1% (Parts I and II). The interassay accuracy at different QC sample concentration levels ranged from 94% to 107%. The acceptance criteria were fulfilled in all accepted batches. The calibration range was 1.43‐215 µg/mL.

The total number of samples analyzed for free valproate was 1747. The interassay CV% for accepted QC samples in the study sample batches was 2.4%‐2.9% (Parts I and II). The interassay accuracy at different QC sample concentration levels ranged from 95% to 104% (Parts I and II). The calibration range was 0.36‐100 µg/mL.

### Pharmacokinetic Analyses

The PK parameters were calculated by standard noncompartmental analysis (NCA) methods using individual plasma concentration–time data from each subject and Phoenix WinNonlin Version 8.3 (Pharsight Corp.) software. An interim evaluation of the PK and safety data from Part I was performed before initiation of Part II. Accumulation indices (R_acc_) were calculated for repeated dosing in Part II as the observed accumulation ratios from the first and last dosing intervals as follows:

$$R_{\mathrm{acc}\_\mathrm{AUCtau}}=\mathrm{AU}C_{\mathrm{tau}-\mathrm{last}}/\mathrm{AU}C_{\mathrm{tau}-1}$$

where AUC_tau‐last_ is the area under the plasma concentration–time curve (AUC) during the last dosing interval and AUC_tau‐1_ is the AUC during the first dosing interval. For observed peak concentrations, the accumulation index R_acc_Cmax_ was defined as

$$R_{\mathrm{acc}\_\mathrm{Cmax}}=C_{\max-\mathrm{last}}/C_{\max-1}$$

where C_max‐last_ is the highest observed concentration during the last dosing interval and C_max‐1_ is the highest observed concentration during the first dosing interval.

### Safety Assessments

Adverse events (AEs) were recorded from the time of informed consent until the last visit. The subjects were monitored for AEs during the study visits, and they were asked about AEs with nonleading questions. All AEs were classified by system organ class and preferred term using the Medical Dictionary for Regulatory Activities coding system Version 24.1, and according to severity and causality.

Clinical safety assessments included physical examinations, vital signs, body weight, 12‐lead ECG, and audiograms. The audiograms captured the hearing level (decibel) results for each of the measured frequencies, that is, 250, 500, 1000, 2000, 4000, 6000, and 8000 Hz, with special emphasis on the speech range (500‐4000 Hz). Safety laboratory samples were analyzed with accredited methods at Turku University Hospital.

### Peripheral Blood Mononuclear Cell Collection and Sample Preparation

Blood samples for PBMC isolation were collected before dosing and 3 times during the treatment period (23.5, 47.5, and 80 hours after the first dose) in Part II of the study. PBMCs were isolated from heparinized whole blood samples with Cell Preparation Tubes (BD Biosciences). The cell pellets were washed and diluted with phosphate‐buffered saline, aliquoted in cryotubes at equal cell counts (0.75‐1.0 × 10^6^) and stored frozen at −80°C.

### Western Blotting

Valproate‐induced increases in histone acetylation in PBMCs were compared after repeated dosing of the test and reference formulations with semiquantitative Western blotting of acetylated histone 3. The modified analysis method^9^ was based on sodium dodecyl sulfate–polyacrylamide gel electrophoresis, semidry transfer to PVDF membranes, immunoblotting, and electrochemiluminescence detection. In brief, the cell pellets were lysed in Laemmli sample buffer containing sodium dodecyl sulfate, β‐mercaptoethanol, and protease and phosphatase inhibitors (Roche); sonicated with the Diagenode Bioruptor system for 10 minutes; heated (+95°C, 5 minutes); and centrifuged +4°C, 5 minutes), and the supernatant was separated into a new tube. Before loading, the samples were reheated (+95°C, 5 minutes), and aliquots representing approximately 0.75 × 10^5^ cells were loaded per well onto precast TGX gels (Bio‐Rad Laboratories). After electrophoresis, the samples were transferred to PVDF membranes using semidry transfer. The transfer quality was ensured with Ponceau S staining, and the membranes were blocked with 5% nonfat milk solution. The primary antibody incubations were performed overnight at +4°C. The secondary antibodies were incubated for 1 hour at room temperature. The following antibodies and dilutions (in 1% nonfat milk solution) were used: rabbit anti–acetylated lysine 9 of histone H3 (acH3K9) antibody, 1:1000 (No. 9649, Cell Signaling Technologies), rabbit anti–glyceraldehyde‐3‐phosphate dehydrogenase (GAPDH) antibody, 1:10 000 (ab181602, Abcam) and HRP‐conjugated anti‐rabbit antibody, 1:10 000 for detection of acH3K9 and 1:3000 for detection of GAPDH (No. 7074, Cell Signaling Technologies). The membranes were developed using electrochemiluminescence reagents (No. 34075, Thermo Fisher), images were captured with a Sapphire imaging system (Azure Biosystems), and the band intensities were quantified using ImageJ software Version 1.53c.^12^ The acH3K9 signal was normalized by dividing the signal for each band by a lane normalization factor obtained on the basis of the loading control GAPDH signal. The normalized acH3K9 band intensity of each time point after dosing was then compared with the predosing sample of each subject to come up with fold changes compared to baseline.

Before study sample analysis, validation experiments were carried out to ensure antibody validity, robustness of the method, and appropriate conditions for signal quantitation (eg, membrane transfer, sample load, antibody dilutions). Repeatability of the assay was tested with 2 sets of samples, with observed CV% for the fold changes from baseline of 8% and 15% (n = 7 for both sets).

### Statistical Analysis

All statistical analyses were done using SAS Version 9.4 (SAS Institute Inc.) or GraphPad Prism Version 10.4 (Dotmatics). All final statistical and PK analyses were done after the statistical analysis plan had been finalized and approved, the Clean File Report including decisions on analysis sets had been signed, and the database had been locked.

## Results

### Study Population

A total of 40 healthy volunteers were enrolled, 13 in Part I and 27 in Part II. Table S1 describes the study population demographics. In Part I, 1 subject discontinued the study after dosing in Period 1 and was replaced. Thus, 12 subjects completed Part I of the study. In Part II, dosing was interrupted earlier than per protocol for 3 participants because of vomiting (1 in each treatment group) or elevated liver enzymes (1 subject in the reference group). Thus, 24 subjects completed the dosing regimen according to the study protocol.

### Pharmacokinetic Results

Plasma concentration data of both total and free valproate were obtained for all subjects included in the PK analysis set.

#### Part I (Single Doses)

**Figure 1:**
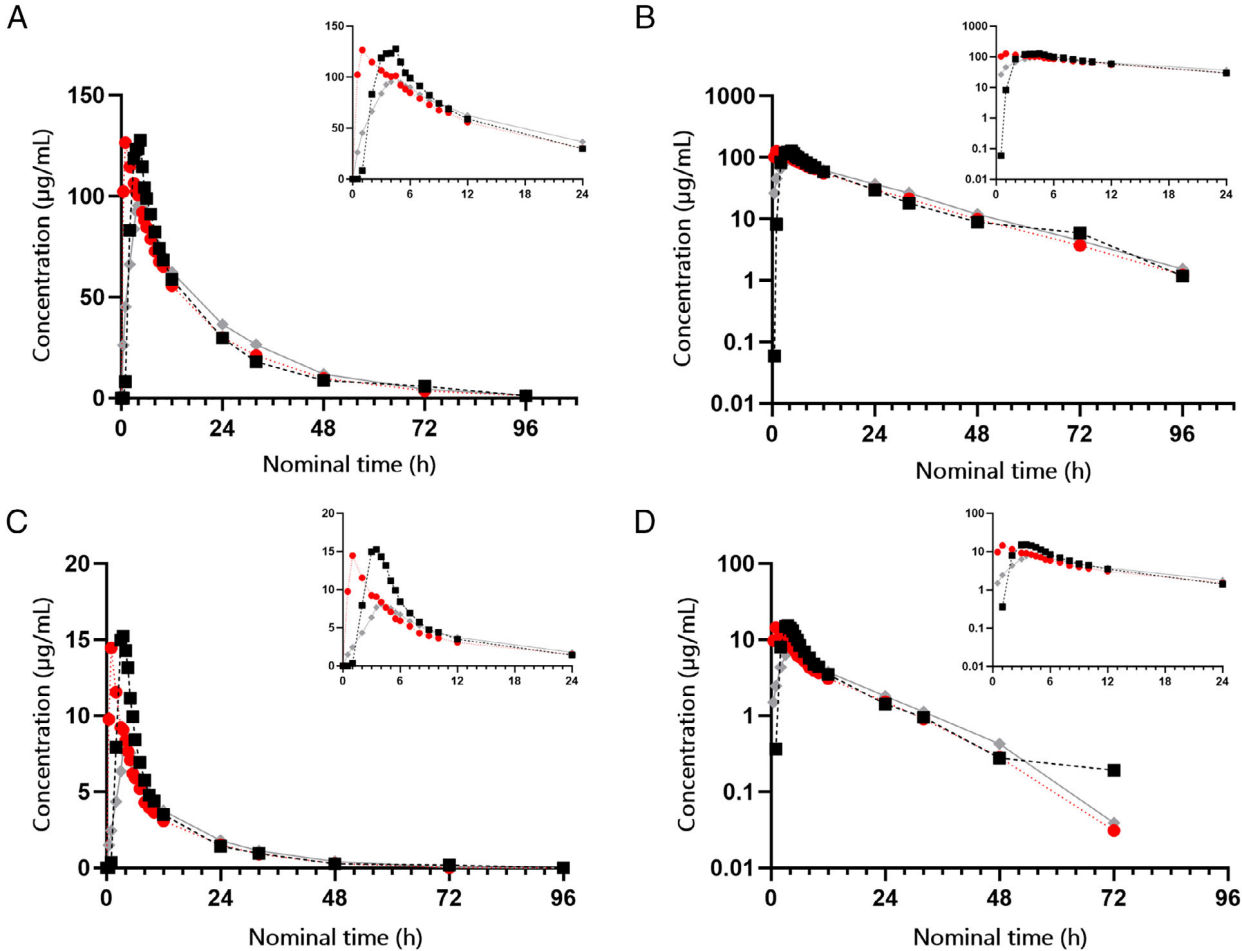
Overview of mean plasma concentrations of valproate (µg/mL) administered as test (fasted), test (fed), and reference (fasted)—Part I. Black symbols = reference; red = test (fasted); gray = test (fed). (A) Linear total plasma concentration–time curves; (B) semilogarithmic total plasma concentration–time curves; (C) linear free plasma concentration–time curves; (D) semilogarithmic free plasma concentration–time curves. Inserts in the panels show the same concentration data on a different time scale, from dose intake until 24 hours after dosing.

The observed concentration profiles of total valproate in plasma showed some differences among the 3 treatments, especially in the initial parts of the plasma curves. The arithmetic mean maximum plasma concentration (C_max_) was lowest for the test product given with food (105 µg/mL), intermediate for the test product given in the fasted condition (128 µg/mL), and highest for the reference product (without food; 145 µg/mL). After C_max_, the concentration curves showed biphasic declines over the 96‐hours observation period (Figure 1A,B). Free drug concentrations followed the total concentration profiles, at approximately 9%‐13% of the corresponding total concentrations (Figure 1C,D). Observed lag times in drug absorption were short in general, but the start of absorption of valproate from the reference formulation showed some delay (median lag time, 0.5 [range, 0‐3] hours). Median time to C_max_ was 1.0 hours for the test formulation in the fasted state, 3.0 hours for the reference formulation in the fasted state and 4.2 hours for the test product given with food. For the 12 subjects who completed the trial, all PK parameters could be derived for the test product, with and without food. For the reference product, PK parameters dependent on the terminal elimination rate constant λ (AUC from time zero to infinity [AUC_0‐inf_]) could not be reliably estimated for 2 subjects for total valproate and for 3 subjects for free valproate because of uncertainty in terminal elimination rate constant λ determination (r^2^ 0.80 or less).

All 3 treatments showed similar elimination profiles, with arithmetic mean terminal elimination rate ranging between 15.8 and 16.3 hours. The plasma exposure of valproate (AUC_0‐inf_) was similar after all 3 treatments. The PK parameters of the 3 single‐dose treatments are summarized in Table 1.

**Table 1:** Overview of PK Parameters per Treatment—Part I

		Total valproate in plasma			Free (protein‐unbound) valproate in plasma		
Parameter (unit)	Summary statistic	Test (fasted)	Test (fed)	Reference (fasted)	Test (fasted)	Test (fed)	Reference (fasted)
t_lag_ (hours)	n	12	12	13	12	12	13
	Median	0.0	0.0	0.5	0.0	0.0	1.0
	Minimum	0.0	0.0	0.0	0.0	0.0	0.5
	Maximum	0.0	0.0	3.0	0.0	0.5	3.0
C_max_ (µg/mL)	n	12	12	13	12	12	13
	Mean	128	105	145	14.9	9.3	19.1
	SD	10.4	10.5	14.4	4.4	1.6	3.7
t_max_ (hours)	n	12	12	13	12	12	13
	Median	1.0	4.2	3.0	1.0	3.5	3.5
	Minimum	0.5	2.0	2.0	0.6	3.0	2.0
	Maximum	2.0	6.0	4.5	2.0	6.0	4.5
t_1/2_ (hours)	n	12	12	11	12	12	10
	Minimum	10.9	11.0	11.5	8.2	9.1	8.2
	Maximum	22.8	21.5	23.4	18.0	18.7	20.0
	Mean	16.3	15.8	15.9	12.4	12.6	10.7
	SD	3.6	3.3	4.0	2.8	2.6	3.5
AUC_0‐inf_ (µg•h/mL)	n	12	12	11	12	12	10
	Mean	2200	2298	2138	133	127	141
	SD	423	532	594	29.4	30.3	42.3
	CV%	19.2	23.1	27.8	22.2	24.0	30.1

AUC_0‐inf_, area under plasma concentration–time curve from time zero to infinity; C_max_, maximum plasma concentration; CV, coefficient of variation; SD, standard deviation; t_1/2_, elimination half‐life; t_lag_, lag time; t_max_, time to maximum plasma concentration.

When administered as single doses under fasted conditions, the 2 formulations of valproate (test and reference) were comparable with regard to AUC_0‐inf_, as seen in Table 1. Bioequivalence was demonstrated for total valproate since the 90% confidence intervals (CIs) for the test/reference ratios of AUC_0‐inf_ (1.01‐1.16) and C_max_ (0.84‐0.92) were within the acceptance range of 0.80‐1.25. For free valproate, the 90% CI of the test/reference ratio for AUC_0‐inf_ was 0.95‐1.11, but for C_max_ of free valproate, bioequivalence was not demonstrated since the observed 90% CI of the ratio, 0.70‐0.86, was outside of the acceptance limits for bioequivalence (0.80‐1.25) (see Table S2). Delayed and higher C_max_ levels were measured for total and free valproate after a single dose of the reference product compared to a single dose of the test product, given under fasted conditions.

The impact of food on the bioavailability of valproate from the test formulation was evaluated by administering it under fed and fasted conditions; the PK results are summarized in Table S3. Bioequivalence was shown for AUC_0‐inf_ of total valproate after administration of the test product under fed and fasted conditions. The 90% CI of the fed/fasted AUC_0‐inf_ ratio (0.97‐1.11) was within the acceptance range of 0.80‐1.25. The result was similar also for free valproate (90% CI of fed/fasted ratio, 0.89‐1.02). In terms of C_max_, bioequivalence was not demonstrated for total and free valproate, since the 90% CIs of the C_max_ ratios (0.79‐0.87 and 0.58‐0.70, respectively) were outside of the acceptance range (0.80‐1.25) (see Table S3).

#### Part II (Multiple Doses)

**Figure 2:**
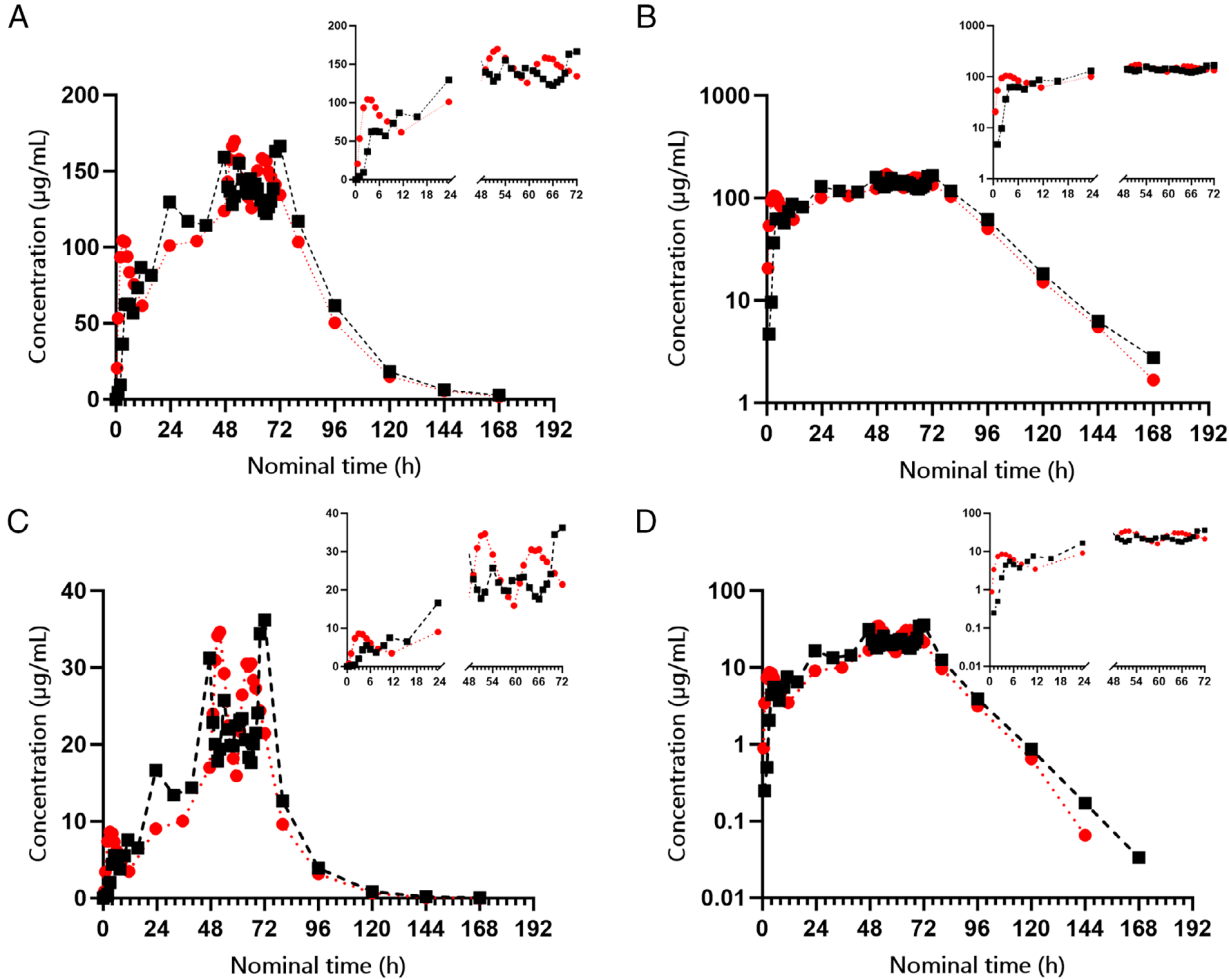
Overview of mean plasma concentrations of valproate (µg/mL) after repeated doses of test or reference, under fed conditions—Part II (PK analysis set). Black symbols = reference; red = test. (A) Linear total plasma concentration–time curves; (B) semilogarithmic total plasma concentration–time curves; (C) linear free plasma concentration–time curves; (D) semilogarithmic free plasma concentration–time curves. Inserts in the panels show the same concentration data over the first and last 24 hours of the 72‐hour dosing period.

When repeated doses of the 2 formulations were administered under fed conditions at approximately equal total daily doses (60 mg/kg/day), although with different doses per administration and different dosing intervals, the 2 formulations resulted in similar steady state levels of total and free valproate in plasma (Figure 2). Reference tablets were administered at a dose of approximately 20 mg/kg every 8 hours for a total of 9 doses (3 days in total), while test product capsules were administered at a dose of approximately 30 mg/kg every 12 hours for 6 doses in total (3 days in total). Average total valproate concentrations over a dosing interval after the last dose were 146 µg/mL for the test product versus 137 µg/mL for the reference product. For free valproate, the corresponding values of concentrations over a dosing interval at steady state were 26.2 and 24.3 µg/mL. Selected PK parameters are listed per treatment in Table 2. All 24 completed subjects could be fully characterized for PK, except for 1 in the reference group who had incomplete PK results regarding free valproate because of inability to reliably estimate the terminal elimination rate constant λ (r^2^ ≤ 0.80).

**Table 2:** Overview of PK Parameters per Treatment—Part II

		Total valproate in plasma		Free (protein‐unbound) valproate in plasma	
Parameter (unit)	Summary statistic	Test	Reference	Test	Reference
C_max‐last_ (µg/mL)	n	12	12	12	12
	Mean	164	171	33	38
	SD	26.3	17.2	13.2	9.2
t_1/2‐last_ (hours)	n	12	12	12	11
	Mean	15.2	15.6	11.5	10.8
	SD	2.2	2.5	2.3	2.8
AUC_(0‐inf)‐last_ (µg•h/mL)	n	12	12	12	11
	Mean	4957	4859	576	551
	SD	975	965	255	198
	CV%	19.7	19.9	44.3	35.9
Fluctuation (%)	n	12	12	12	12
	Mean	23.2	47.0	51.5	106
	SD	8.1	28.2	19.8	57.8
R_acc_AUCtau_	n	12	8	12	8
	Mean	1.9	2.9	4.8	7.6
	SD	0.2	0.8	1.2	3.3
R_acc_Cmax_	n	12	12	12	12
	Mean	1.5	2.9	3.4	11.5
	SD	0.2	1.8	0.9	11.9
C_av_ (µg/mL)	n	12	12	12	12
	Mean	146	137	26.2	24.3
	SD	25.0	18.9	11.5	6.7

AUC_(0‐inf)‐last_, area under the plasma concentration–time curve from the last dose to infinity; C_av_, average concentration during a dosing interval at steady state; C_max‐last_, maximum plasma concentration after the last dose; CV, coefficient of variation; t_1/2‐last_, elimination half‐life after the last dose; R_acc_AUCtau_, accumulation index based on area under the plasma concentration–time curve over a dosing interval; R_acc_Cmax_, accumulation index based on maximum plasma concentration; SD, standard deviation.

Also, C_max_ at steady state (ie, C_max_ after the last dose) was comparable for both total and free concentrations of valproate between the 2 formulations, with means of 164 µg/mL for total and 33 µg/mL for free valproate after repeated doses of the test formulation and 171 µg/mL for total and 38 µg/mL for free valproate after repeated doses of the reference product. The drug exposure at steady state, that is, AUC_(0‐inf)‐last_ was comparable between the 2 formulations, as seen in Table 2. The accumulation of valproate (R_acc_AUC_ and R_acc_Cmax_) and the observed fluctuation of plasma concentrations were greater for the reference product compared to the test product. It should be noted that the estimate of fluctuation (peak‐to‐trough variation) is here based on C_min_ (defined as the minimum observed concentration during a dosing interval) which was the predefined parameter in the clinical study protocol. The average extent of fluctuation in total valproate concentrations was 23.2% for the test product and 47.0% for the reference product (*P* = .0102, *t* test).

### Adverse Events

No serious or severe AEs were reported in the trial. No AEs led to discontinuation from the study or withdrawal from study treatment in Part I; in Part II, 2 subjects were discontinued from study treatment because of vomiting, 1 in each treatment group, and dosing was interrupted in 1 participant in the reference group because of a transient elevation of liver enzymes; this subject received only 5 of the planned 9 doses.

All treatment‐emergent AEs in Part I, apart from one, were of mild intensity; 1 headache was considered to be of moderate intensity. In Part I, headaches were the most common treatment‐emergent AEs. Four of the 10 events of headache were assessed as possibly related to drug intake. Three subjects reported nausea after drug intake, and 1 participant vomited after intake of the reference product. This participant thereafter discontinued his participation in the study for “personal reasons.”

No apparent differences were identified in Part II between the 2 treatment groups in terms of the number or intensity of AEs. There were slightly more AEs that were considered causally related to the study drug in the group receiving the test product (51 events compared to 37 in the reference group). This was mainly driven by reports of nausea (17 related events in the test product group compared to 9 in the reference group), dyspepsia (7 related events after the test product and 1 after the reference product) and headache (7 related events in the test formulation group compared to 3 in the reference group). Most AEs were of mild intensity, and all were resolved by the end of the study.

Apart from transiently elevated liver enzymes observed in 1 participant on Day 2 of repeated dosing of the reference product, there were no clinically significant abnormalities reported for any of the laboratory parameters measured or for parameters of physical examination, vital signs, or ECG.

As valproate has been associated with auditory side effects, such as reversible sensorineural hearing loss and tinnitus,^7^, ^8^, ^10^ special attention was paid to possible auditory symptoms, and audiograms were recorded at screening and before the end‐of‐study visit. In Part I, 2 events of transient tinnitus were reported, 1 after the test product (without food) and 1 after the reference product; no changes were noted in the audiograms. In Part II, transient subjective hypoacusis was reported by 1 subject in the test formulation group, and 2 subjects, 1 in each treatment group, exhibited transient sensorineural hearing loss in an audiogram recorded approximately 2 weeks after the last dose. These subjects were invited for repeated audiogram evaluations after another 4 weeks. All auditory AEs were resolved in the repeated audiograms.

### Histone Acetylation in PBMCs

**Figure 3:**
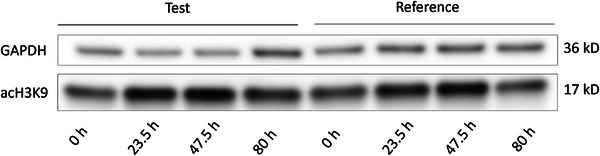
Representative Western blot showing immunoreactive bands representing GAPDH (loading control) and histone 3 acetylated at lysine 9 (acH3K9) in the upper and lower panels, respectively. The first 4 lanes from the left represent samples from a subject in the test group, and the last 4 are from a subject in the reference group, at respective time points. Molecular masses are indicated on the extreme right, and sampling time points (from the start of dosing) at the bottom.

Acetylation of lysine 9 of histone H3 (acH3K9) in PBMCs was measured using Western blotting in order to evaluate the extent of treatment‐induced inhibition of histone deacetylation. Evidence of inhibition of histone deacetylase activity, that is, increased levels of acH3K9, was observed after administration of both products (Table 3; *P* = .004 for effect of time, 2‐way analysis of variance; the difference between treatments was not statistically significant; *P* = .30 for the time × treatment interaction; see Figure 3 for a representative Western blot image). The largest increases in histone acetylation (69%) were observed at approximately 48 hours after the start of dosing of the test product. Variability in the observed responses was quite marked.

**Table 3:** Fold Changes From Baseline in Histone Acetylation in PBMCs After 3 Days of Treatment With 2 Valproate Formulations

	Test formulation (n = 12)		Reference formulation (n = 12)	
Time (hours)	Mean (SD)	CV%	Mean (SD)	CV%
23.5	1.44 (0.51)	36	1.32 (0.48)	36
47.5	1.69 (0.79)	47	1.29 (0.34)	26
80	1.26 (0.74)	59	0.98 (0.33)	34

“Time” indicates the PBMC sample collection time point after intake of the first dose of study medication.

Both drugs were dosed orally at ≈60 mg/kg/day. Acetylation of lysine 9 of histone H3 was measured using Western blotting.

CV, coefficient of variation; PBMC, peripheral blood mononuclear cell; SD, standard deviation.

## Discussion

The PK results and valproate concentrations obtained in the current study can now be compared with the results of the previous Phase I/IIa trial^10^ where only sparse blood sampling was performed for determination of valproate concentrations. Based on the comparable valproate exposure demonstrated between the 2 formulations in the current study, the efficacy results of the previous patient study, where the patients were administered enteric‐coated immediate‐release valproate tablets at the same dose and dosing frequency as in the current study, can be bridged and considered to be valid also for the test formulation.

When the 2 formulations, test and reference, were administered as single doses under fasted conditions, they resulted in equivalent exposure (AUC) for total and free valproate and had quite similar PK characteristics, apart from the faster start of absorption from the test product. The formulations were also similar in terms of the proportion of free valproate that was available in plasma in relation to total valproate (9%‐13%). The delayed absorption of the reference product, observed as a lag time, is explained by the protective coating of the tablets, causing delayed dissolution and release, whereas the test product, consisting of 75% of immediate‐release granules of sodium valproate, allows for faster dissolution and consequently earlier start of absorption of the active ingredient.^13^

The effect of food intake on the absorption of valproate from the new test formulation was also investigated, since drug intake with food is recommended for the reference product in order to avoid gastrointestinal adverse effects. Administration of the test product after a high‐fat, high‐calorie meal resulted, as expected, in delayed absorption and lower C_max_ values for both total and free valproate compared to dosing in the fasted state. Nevertheless, bioequivalence (ie, no food effect) was confirmed in terms of drug exposure (AUC_0‐inf_) for both total and free valproate. Intake of the test product with food may thus be considered beneficial, decreasing the peak concentrations, with lesser risk of adverse effects in general and especially gastrointestinal side effects, but still maintaining similar exposure as under fasted conditions.

In the PK analysis following multiple dosing of both formulations under fed conditions, the 2 formulations provided similar steady state exposure (AUC from the last dose to infinity), with comparable concentrations of C_max_ after the last dose for both total and free valproate. This was despite different numbers of daily doses and different dosing intervals. Still, the fluctuation of plasma drug concentrations was significantly less with the twice‐daily dosing regimen of the test product and exhibited smaller variation compared to the thrice‐daily dosage regimen of the reference product.

Overall, both formulations of valproate were well tolerated in the current study setting. No serious or severe AEs were reported. The most commonly registered AEs were nausea and headache, both of which have previously been reported in relation to valproate. In combination cancer treatment, it is possible that these symptoms will be alleviated by the concomitant use of glucocorticoids. Hearing impairment is another AE that has previously been reported in association with intake of high doses of valproate.^10^ In the current study, there were 3 cases of transiently impaired hearing. All 3 events of hearing impairment were recovered in follow‐up audiograms.

In summary, even if the study was conducted in healthy subjects and not in the intended patient population, the results support the safe administration of the test product at approximately 30‐mg/kg doses of sodium valproate taken with food twice daily for 3 consecutive days. A favorable PK profile was observed considering the twice‐daily dosing regimen of the test product compared with the thrice‐daily dosing regimen of the reference product, when both formulations were given at the same total daily dose of approximately 60 mg/kg. This further supports the positive efficacy results previously observed in patients with DLBCL.^10^ Specifically, in the current study, the 2 formulations were bioequivalent for valproate in terms of plasma exposure, but the test product was associated with lower C_max_ values of valproate, which can be interpreted as a beneficial characteristic from a safety point of view, especially considering the high valproate concentrations in plasma that are needed for the intended indication and inhibition of HDAC activity. The possibility to treat patients with DLBCL with a formulation of valproate at fewer daily doses, that is, 2 daily doses instead of 3 daily doses of conventional enteric‐coated tablets, may be considered an advantage, as many individuals in the intended patient population may be in poor health and have severe symptoms related to both their disease and a challenging treatment regimen. Obtaining relevant and predictable exposure to valproate, with somewhat decreased peak concentrations and with fewer doses to be taken per day, could result in improved treatment adherence and better treatment outcomes in the vulnerable patient population for which the test formulation is intended.

## Conclusions

When 2 sodium valproate formulations, the test and reference products, were administered to healthy subjects as single doses (30 mg/kg) under fasted conditions, they resulted in equivalent exposure (AUC) for total and free valproate. Administration with a meal delayed the absorption of valproate from the test formulation and was associated with a lower C_max_, but exposure to valproate (AUC) was not impacted by the high‐fat, high‐calorie meal.

Following multiple dosing under fed conditions, the 2 formulations produced similar steady‐state exposure, with comparable peak concentrations for both total and free valproate. This was despite the different numbers of daily doses and the different dosing intervals. The fluctuation in plasma concentrations associated with the twice‐daily dosing regimen of the test product was smaller and less variable between individuals compared to the thrice‐daily dosage regimen of the reference product. The current results may thus indicate possible clinical benefits from using the novel capsule formulation containing a combination of immediate‐release granules and extended‐release pellets, compared to conventional sodium valproate enteric‐coated tablets.

## Conflicts of Interest

Mia Lundblad was an employee of Respiratorius AB, which is the parent company of Valcuria AB, at the time of the study conduct. Kristina Drott is a board member of Respiratorius AB and associate professor at the Department of Oncology, Lund University. Elsa Lilienberg is an employee of SDS Life Science AB, Sweden (part of Cytel Inc.). Nikhil Ahuja, Susanna Kääriäinen, Zsófia Lovró, and Mika Scheinin are employees of Clinical Research Services Turku, CRST Oy. Marica T. Engström and Karla Saukkonen are employees of the University of Turku, Finland. CRST Oy, the University of Turku, and SDS Life Science AB were contracted by Valcuria AB to perform the study.

## Funding

The study was funded by Valcuria AB, Lund, Sweden.

## Supporting information



Supporting Information

## Data Availability

The data will not be freely available for sharing at this time as the data are commercially confidential. However, Valcuria AB will consider reasonable requests to share the data.
