# Supplementary material for: Combined Immediate‐Release and Extended‐Release Formulation of Sodium Valproate Provides Stable Plasma Levels for Inhibition of Histone Deacetylation
Source: Clin Pharmacol Drug Dev. 2025 May 27;14(9):717–27. doi: 10.1002/cpdd.1555 (PMC12402831; doi:10.1002/cpdd.1555)
Supplement: Supplementary file 1 — Supporting Information [file CPDD-14-717-s001.docx]

Combined immediate-release and extended release formulation of sodium valproate provides stable plasma levels for inhibition of histone deacetylation

Nikhil Ahuja^1^, Susanna Kääriäinen^1^, Zsófia Lovró^1^, Mia Lundblad^2^, Kristina Drott^2^, Elsa Lilienberg^3^, Marica T. Engström^4^, Karla Saukkonen^4^, Mika Scheinin^1, 4^

1 Clinical Research Services Turku, CRST Oy, Finland

2 Valcuria AB (wholly owned subsidiary of Respiratorius AB), Sweden

3 SDS Life Science AB (part of Cytel Inc.), Sweden

4 Institute of Biomedicine, University of Turku, Finland

Supplementary tables

Table S1. Summary of population demographics

|  |  | PART I | | | | PART II | | |
| --- | --- | --- | --- | --- | --- | --- | --- | --- |
| Variable |  | ACB (n=4) | BAC (n=4) | CBA (n=5) | Total (n=13) | Test (n=13) | Reference (n=14) | Total (n=27) |
| Sex | Female | 1 (25.0%) | 1 (25.0%) | 2 (40.0%) | 4 (30.8%) | 3 (23.1%) | 3 (21.4%) | 6 (22.2%) |
|  | Male | 3 (75.0%) | 3 (75.0%) | 3 (60.0%) | 9 (69.2%) | 10 (76.9%) | 11 (78.6%) | 21 (77.8%) |
| Age | Mean (SD) | 39.5 (18.7) | 46.5 (15.8) | 42.6 (14.5) | 42.8 (15.1) | 39.0  (13.2) | 40.0 (16.9) | 39.5 (15.0) |
| Weight (kg) | Mean (SD) | 83.0 (7.6) | 78.8 (13.8) | 83.1 (8.5) | 81.7 (9.5) | 76.2 (8.2) | 79.5 (12.9) | 77.9 (10.8) |
| BMI (kg/m2) | Mean (SD) | 25.2 (2.6) | 25.6 (3.4) | 27.3 (3.8) | 26.1 (3.2) | 25.0 (3.1) | 25.8 (2.9) | 25.4 (3.0) |

Abbreviations: BMI = body mass index, n = number of subjects, SD = standard deviation

Table S2. Relative bioavailability of valproate evaluated on the basis of area under the plasma concentration-time curve (AUC) and observed maximum concentration in plasma (C_max_). Modified controlled-release capsules (Test formulation) and conventional enteric-coated tablets (Reference formulation) were given in the fasted state (formulation effect in the fasted state) - Part I (n = 11-13)

|  | | | | | Comparison of Test vs. Reference | |
| --- | --- | --- | --- | --- | --- | --- |
|  | PK parameter (unit) | Treatment | n | GLSM | Ratio | 90% CI |
| Total valproate | AUC_(0-inf)_ (h*µg/mL) |  |  |  | 1.08 | 1.01 - 1.16 |
|  |  | Test | 12 | 2212 |  |  |
|  |  | Reference | 11 | 2046 |  |  |
|  | AUC_(0-t)_ (h*µg/mL) |  |  |  | 1.08 | 1.01 - 1.14 |
|  |  | Test | 12 | 2147 |  |  |
|  |  | Reference | 13 | 1994 |  |  |
|  | C_max_ (µg/mL) |  |  |  | 0.88 | 0.84 - 0.92 |
|  |  | Test | 12 | 128 |  |  |
|  |  | Reference | 13 | 145 |  |  |
| Free valproate | AUC_(0-inf)_ (h*µg/mL) |  |  |  | 1.03 | 0.95 - 1.11 |
|  |  | Test | 12 | 134 |  |  |
|  |  | Reference | 10 | 130 |  |  |
|  | AUC_(0-t)_ (h*µg/mL) |  |  |  | 0.95 | 0.86 - 1.04 |
|  |  | Test | 12 | 123 |  |  |
|  |  | Reference | 13 | 130 |  |  |
|  | C_max_ (µg/mL) |  |  |  | 0.78 | 0.70 - 0.86 |
|  |  | Test | 12 | 14.6 |  |  |
|  |  | Reference | 13 | 18.7 |  |  |

AUC_(0-inf)_ = area under the plasma concentration-time curve from time zero extrapolated until infinity; AUC_(0-t)_ = area under the concentration-time curve from time zero to time of last measurable concentration; CI = confidence interval; C_max_ = observed maximum concentration in plasma; GLSM = geometric least squares mean; n = number of observations

Table S3. Relative bioavailability of valproate evaluated on the basis of area under the plasma concentration-time curve (AUC) and observed maximum concentration in plasma (C_max_). Modified controlled-release capsules (Test formulation) were given with (fed) and without food (fasted) – Part I (n = 12).

|  |  | | | Comparison of fed vs. fasted | |
| --- | --- | --- | --- | --- | --- |
|  | PK parameter (unit) | Treatment | GLSM | Ratio | 90% CI |
| Total valproate | AUC_(0-inf)_ (h*µg/mL) |  |  | 1.04 | 0.97 - 1.11 |
|  |  | Fed | 2292 |  |  |
|  |  | Fasted | 2212 |  |  |
|  | AUC_(0-t)_ (h*µg/mL) |  |  | 1.03 | 0.97 - 1.10 |
|  |  | Fed | 2222 |  |  |
|  |  | Fasted | 2147 |  |  |
|  | C_max_ (µg/mL) |  |  | 0.83 | 0.79 - 0.87 |
|  |  | Fed | 105 |  |  |
|  |  | Fasted | 128 |  |  |
| Free valproate | AUC_(0-inf)_ (h*µg/mL) |  |  | 0.95 | 0.89 - 1.02 |
|  |  | Fed | 127 |  |  |
|  |  | Fasted | 134 |  |  |
|  | AUC_(0-t)_ (h*µg/mL) |  |  | 0.94 | 0.86 - 1.04 |
|  |  | Fed | 116 |  |  |
|  |  | Fasted | 123 |  |  |
|  | C_max_ (µg/mL) |  |  | 0.64 | 0.58 - 0.70 |
|  |  | Fed | 9.3 |  |  |
|  |  | Fasted | 14.6 |  |  |

AUC_(0-inf)_ = area under the plasma concentration-time curve from time zero extrapolated until infinity; AUC_(0-t)_ = area under the concentration-time curve from time zero to time of last measurable concentration; CI = confidence interval; C_max_ = observed maximum concentration in plasma; GLSM = geometric least squares mean

Supplementary figure


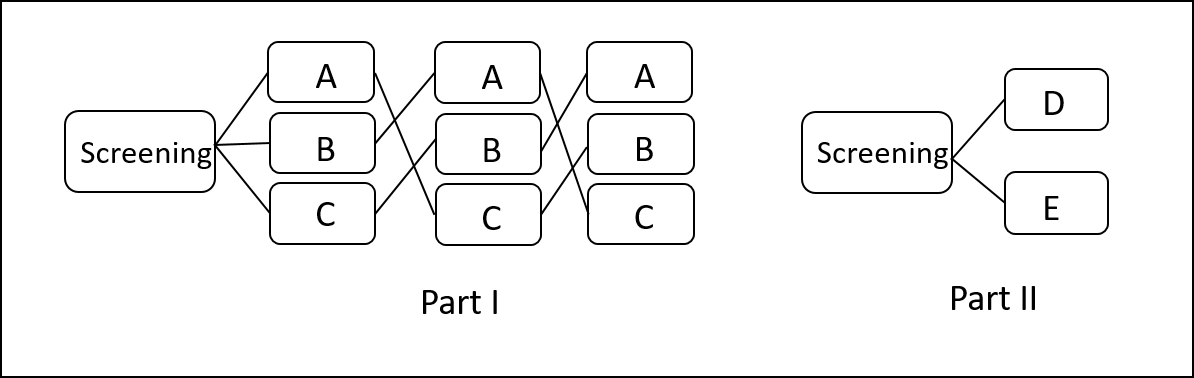


Figure S1. Schematic presentation of the study design: Part I followed a 3-treatment, 3-period cross-over design. Treatments A and C represent Test and Reference under fasted conditions and B represents Test intake after a high-fat high-calorie meal. In Part II, participants received multiple doses of either Test (D) or Reference (E) under fed conditions over a period of 3 days.
